# Microglia and astrocyte responses to neuropathogenic protozoan parasites

**DOI:** 10.12703/r/10-69

**Published:** 2021-09-03

**Authors:** Azadeh Nasuhidehnavi, George S Yap

**Affiliations:** 1 Center for Immunity and Inflammation, New Jersey Medical School, Rutgers, The State University of New Jersey, Newark, NJ, USA

**Keywords:** Cerebral malaria, cerebral toxoplasmosis, microglia, astrocytes, alarmin, neuroinflammation

## Abstract

Cerebral toxoplasmosis and cerebral malaria are two important neurological diseases caused by protozoan parasites. In this review, we discuss recent findings regarding the innate immune responses of microglia and astrocytes to *Toxoplasma* and *Plasmodium* infection. In both infections, these tissue-resident glial cells perform a sentinel function mediated by alarmin crosstalk that licenses adaptive type 1 immunity in the central nervous system. Divergent protective or pathogenic effects of type 1 activation of these astrocytes and microglia are revealed depending on the inherent lytic potential of the protozoan parasite.

## Introduction

Neurological diseases associated with neuroinflammation have an autoimmune, neurodegenerative, or infectious etiology^1^. Although central nervous system (CNS) infections caused by viral and bacterial pathogens have been better studied, neurotropic protozoan parasites also cause two major diseases, namely cerebral toxoplasmosis (CT) and cerebral malaria (CM). CT arises as a result of invasion of the CNS cells by *Toxoplasma gondii* parasites that exist in both lytic (tachyzoites) and latent (bradyzoites) forms. In immunocompromised patients, life-threatening necrotic encephalitis stems from a failure to control the lytic stage of the parasite. In immunocompetent individuals, *T. gondii* establishes a chronic infection that has been linked to several neuropsychiatric disorders such as anxiety, suicidal behaviors, and schizophrenia^2–4^. Unlike in CT, there is no threat of parasite replication in neural cells in CM, and neuropathological symptoms occur acutely. It is thought that occlusion of cerebral microvasculature by *Plasmodium*-infected red blood cells (iRBCs) causes increased blood–brain barrier (BBB) permeability, hemorrhage, and induction of cerebral hypoxia^5^. In a mouse model of experimental CM (ECM), inflammation caused by sequestered inflammatory leukocytes, including monocytes and CD8^+^ T cells, also contributed to the development of secondary neurological symptoms^6,7^. CM often appears as a severe and lethal neurological complication, but even after recovery, long-term neurological deficits such as cognitive and behavioral disorders persist in children with complicated malaria^8^.

Here, we discuss the most recent findings regarding the immune responses of two major CNS glial cells—microglia and astrocytes—during *Toxoplasma* and *Plasmodium* infection. In both CT and CM, microglia and astrocytes mount a shared T helper 1 (T_H_1) program of pro-inflammatory and anti-inflammatory cytokine cascades that have divergent protective and pathogenic effects largely dictated by the inherent lytic threat posed by the pathogen to the tissue integrity of the CNS. The mechanistic studies discussed in this short review largely use established mouse models to CT and CM that recapitulate key pathogenetic and clinical features of their respective human diseases.

## Microglia responses in cerebral toxoplasmosis and cerebral malaria

Microglia are CNS-resident immune cells originating from yolk sac myeloid progenitors with multifaceted functions during physiological and pathological conditions^9^. Microglia constantly monitor their microenvironment and respond to pathogenic infections exhibiting reactive phenotypes and immune responses^10^. Moreover, engagement of microglia and astrocytes in the neurovascular unit allows the initiation of glial effector functions in response to BBB injury^11^. Several lines of evidence demonstrated microglia activation during *Toxoplasma* and *Plasmodium* infection. *In vitro* studies showed that upon exposure to *T. gondii* tachyzoites and *Plasmodium*-iRBCs, microglia underwent morphological changes and upregulated the expression of inflammatory markers^12,13^. An important question is whether the microglial phenotype is modulated through direct effects of the parasites or exposure to inflammatory mediators. Bhandage *et al*. revealed that *T. gondii–*infected microglia displayed a hypermotile phenotype dependent on the presence of live parasites in the cells^14^. Microglial hypermigratory behavior is mediated by autocrine GABAergic signaling in *T. gondii–*infected cells^14^. However, these data were derived from *in vitro* studies, and the behavior of infected microglia *in vivo* needs to be investigated.

In contrast to *T. gondii* infection, *Plasmodium*-iRBCs do not directly interact with microglia. Instead, hemodynamic alterations resulting from adherence of inflammatory leukocytes to postcapillary venule endothelium lead to leakage of plasma into the postcapillary space^15^. Exposure of microglia to inflammatory milieu in the postcapillary space may lead to their activation^15^. In addition, oxygen deprivation may provide additional cues for microglial activation^16^. Collectively, microglial activation can stem from direct interaction with the parasites or changes in the microenvironment surrounding the tissue harboring parasitic lesions.

A hallmark of microglial activation is their acquisition of a pro-inflammatory phenotype. The interleukin 1 (IL-1) family is one of the important groups of pro-inflammatory cytokines upregulated in reactive microglia during several neurodegenerative diseases^17,18^. The role of microglial IL-1 expression has been investigated in CT and CM. A recent study from the Harris lab indicates that IL-1α signaling is critical for the interplay between CNS cells during CT^19^. Using RNA sequencing, transgenic mice, and *ex vivo* cytokine assays, the authors showed that microglia was the major source of IL-1α in *T. gondii*–infected brain^19^. Moreover, IL-1α plays a critical role in the recruitment of immune cells and consequently restriction of parasite growth. The protective effect of microglial IL-1α is mediated by upregulating expression of adhesion molecules on endothelial cells^19^.

Though clearly protective in CT, microglial IL-1 has potential adverse effects on neuronal function in ECM. Reverchon *et al*. showed that induction of microglial IL-1β during ECM was associated with impairment of memory and learning^20,21^. Interestingly, this microglial IL-1 response appears to be driven by IL-33 produced by astrocytes and oligodendrocytes^20,21^. IL-1 itself enhanced IL-33 production by oligodendrocytes, suggesting a potent synergistic crosstalk between microglia and oligodendrocytes mediated by alarmins that may augment the neuroinflammatory responses and neurological symptoms during ECM. Although these results suggest that the reduction of endogenous IL-33 may be a therapeutic target for ECM and alleviates neuronal damage, other studies show that exogenous administration of IL-33 in the early stages of ECM has protective outcomes. IL-33 treatment with or without coadministration of antimalaria drugs results in a reduction of cerebral lesions and amelioration of neuropathological symptoms^22,23^. The protective effects of exogenous IL-33 were associated with T_H_2 cell polarization, regulatory T-cell response and decreased inflammasome activation in microglia, and most likely a reduction in destructive T_H_1 response^22,23^.

Tumor necrosis factor alpha (TNF-α) is another major pro-inflammatory cytokine contributing to the development of CT and CM pathogenesis. In CT, TNF-α signaling has a protective role as it is crucial for microglial production of nitric oxide and restriction of parasite growth^24,25^. Conversely in ECM, it appears that TNF-α signaling is detrimental as it mediates intercellular adhesion molecule 1 (ICAM-1) upregulation in brain endothelial cells and leukocyte sequestration^26^. *In vitro* studies showed that TNF-α was upregulated in microglia during *T. gondii* infection and *Plasmodium*-derived extracellular vesicle/iRBC stimulation^13,27,28^. In addition, microglial TNF-α level is increased in *T. gondii*- and *P. berghei* ANKA (PbA)-infected mice^29,30^. Deckert-Schlüter *et al*. reported that TNF-α induction in microglia during CT was dependent on signals downstream of the interferon gamma (IFN-γ) receptor^31^. Depletion of CD8^+^ T cells, a major source of IFN-γ, results in impaired production of microglial cytokines, including TNF-α in *T. gondii*–infected brain, highlighting a regulatory role for T cells and IFN-γ signaling on microglia cytokine production^32^. Furthermore, production of IFN-γ by microglia themselves^33^ may serve as an autocrine signal for TNF-α production.

Given the potent effects of TNF-α on CNS physiology, such as the regulation of synaptic activity^34^, and CNS remyelination^35^, it will be important to interrogate the effects of increased levels of TNF-α on neuronal and astroglia functions during CT and CM. For example, impairment of neurotransmitter uptake occurs in TNF-α–activated astrocytes^36^ and may result in excessive neuronal excitatory stimulation and death. Moreover, neutralization of TNF-α in IL-10–deficient mice infected with *Plasmodium chabaudi* reduces astrocyte activation and disease severity^37^.

The phagocytic function of microglia represents an important aspect of the intimate microglia–neuron interaction, as it is crucial for elimination of undesired synapses and apoptotic neurons^38^. A recent study by Carrillo *et al*. showed that chronic CT caused the loss of perisomatic inhibitory synapses and ensheathment of neurons by activated microglia in hippocampus and neocortex^39^. Li *et al*. further advanced our understanding about molecular mechanisms underlying microglia–neuron interaction during CT^40^. The authors reported that degenerating neurons marked by complement proteins (C1q and C3) and elevated levels of fractalkine chemokine (CX3CL1) were surrounded by activated microglia. As the fractalkine receptor (CX3CR1), among CNS cells, is exclusively expressed on microglia^41^, fractalkine signaling may regulate the recruitment of microglia to the site of tissue injury and initiate microglia–neuron communication during CT. Furthermore, the presence of complement protein deposits on damaged neurons makes them susceptible to clearance by surrounding microglia^40^. Collectively, these findings suggest that destruction of inhibitory synapse and neuronal structures by phagocytic microglia may cause neuronal dysfunction leading to the emergence of neurological problems.

## Astrocyte responses in cerebral toxoplasmosis and cerebral malaria

Astrocytes, the most abundant glial cell type in the CNS, constitute a heterogenous cell population that maintains neuronal homeostasis. Astrocytes perform a plethora of functions, including regulation of energy metabolism^42^, supporting synaptic structure and plasticity^43,44^, and maintenance and regulation of the BBB^45^.

Disruption of the BBB integrity is a critical step in the pathogenesis of CM leading to brain edema that damages neuronal structure^46^. Medana *et al*. reported that retinal astrocytes exhibited an uneven distribution and ensheathment of retinal blood vessels before the expression of neurological signs^47^. Furthermore, in the late stages of ECM, retinal astrocytes lost their contact with the blood–retinal barrier (BRB)^47^. These findings suggest that loss of astrocytic support on BRB may be involved in the BRB compromise, hemorrhage, and edema.

Astrocytes respond to noxious insults through rapid morphological changes that contain and restrict the spread of tissue injury. Upregulation of the astrocytic cytoskeletal glial fibrillary acidic protein (GFAP) and the subsequent reactive astrogliosis limit pathogen distribution in the CNS^48^. Activated astrocytes with increased GFAP levels have been observed in the brain during chronic CT^49^ and ECM^13^. The protective role of GFAP upregulation during CT was demonstrated by Stenzel *et al*.^49^. The authors showed that GFAP knockout (KO) mice infected with *T. gondii* were unable to control parasite growth and confine inflammatory lesions caused by the parasite^49^. These findings emphasize that activation of astrocytes is critical for a strong protective anti-*Toxoplasma* response. It is not yet clear whether GFAP upregulation is associated with protective or detrimental outcomes in CM. Mice lacking both astrocytic intermediate filaments—GFAP and vimentin—have been shown to exhibit impaired astrocyte activation and larger infarct size after ischemic brain injury^50^. Given the presence of sequestered iRBCs, occlusion of the BBB, and induction of hypoxia described in ECM, it is critical to investigate the role of astrocyte reactivity in this disease.

Astrocytes also mount diverse immunological responses, including production of specific pro- and anti-inflammatory mediators during CT and ECM. IFN-γ plays a critical role in the destruction of *T. gondii* in the host cells through different mechanisms^51^. One of the mechanisms is the activation of signal transducer STAT1, which induces transcription of IFN-γ–dependent genes such as *IRF-1*^52^. Using transgenic mice with specific deletion of STAT1 in astrocytes, Hidano *et al*. showed that a more severe form of CT developed with a higher mortality and cerebral parasite load, suggesting that the astrocytic IFN-γ signaling response to the parasite has protective outcomes^53^. Moreover, loss of STAT1 signaling in astrocytes caused a shift in parasite tropism from neurons to astrocytes as higher numbers of parasite cysts appeared in STAT1 KO astrocytes^53^. The protective function of astrocytes in response to IFN-γ and clearance of parasites could be one explanation of why there are no *T. gondii* astrocytic cysts in wild-type (WT) mice*.* IFN-γ production during ECM, in contrast to CT, contributes to cerebral increase of *Plasmodium* biomass since IFN-γ–deficient mice exhibit lower parasite loads and iRBCs in the brain^54^. Of note, IFN-γ produced by CD4^+^ T cells induces expression of CXCL9 and CXCL10 in cerebral endothelial cells, leading to firm attachment of T cells to brain vasculature and ultimately infiltration of cytotoxic CD8^+^ T cells^55–57^. Although the sources of CXCL9 and CXCL10 during ECM have not been completely determined, astrocytes may represent a potential source for these chemokines as PbA-iRBC–stimulated astrocytes displayed increased levels of CXCL10^13^.

The signaling pathway downstream of glycoprotein 130 (gp130), the ubiquitous signal transducer for members of the IL-6 cytokine family, plays a critical role in the establishment of protective and detrimental responses during CT and ECM. IL-6 signaling regulates various functions in astrocytes, such as activation of JAK/STAT3 signaling pathway, regulation of cell proliferation, and expression of GFAP and vimentin^58,59^. It has been demonstrated that IL-6 is required for the restriction of *T. gondii* proliferation in the brain and the development of protective response against CT^60^. *In vivo* deletion of astrocytic gp130 resulted in reduced astrocyte activation leading to impaired parasite confinement and ultimately the development of extensive necrotic lesions^61^. Conversely, in ECM, IL-6 production has been associated with neurotoxic sequelae. Administration of anti-IL-6 neutralizing antibodies in PbA-infected mice resulted in reduced astrocyte activation associated with decreased glial nodules, neuronal death, and longer survival^62^, suggesting neurotoxic roles of astrocytes driven by IL-6 signaling in ECM.

IL-33 is released from necrotic cells as a cellular alarmin and interacts with its receptor, the orphan IL-1 receptor family member ST2. It has been demonstrated that ST2 is upregulated during CT in the brain, and IL-33/ST2 signaling is found to prevent *T. gondii* growth and decrease neural tissue destruction^63^. Still *et al*. showed that oligodendrocytes and astrocytes were the major sources of IL-33 in *T. gondii*–infected brain^64^. Importantly, IL-33 signals on astrocytes through ST2 receptor leading to the production of inflammatory chemokines such as CCL2 and CXCL10 and infiltration of leukocytes which results in the control of infection^64^. As discussed above, astrocytes in addition to oligodendrocytes also produce IL-33 during ECM^20^. In turn, IL-33 production by astrocytes likely leads to enhanced synaptic engulfment by microglia^65^ and increases the formation of excitatory synapses by neurons^66^. Therefore, it will be important to know how synapse structure and neuronal function are dysregulated by IL-33 produced during both CT and ECM, even though IL-33 has a clear protective effect by controlling *Toxoplasma* infection in the brain.

Transforming growth factor beta 1 (TGF-β1) is a pleiotropic cytokine that negatively regulates immunopathological responses during neuroinflammatory disorders. Cekanaviciute *et al*. reported that TGF-β1 signaling with anti-inflammatory effects was induced in astrocytes in response to *T. gondii* infection^67^. That study showed that *in vivo* inhibition of astrocytic TGF-β1 signaling did not affect *T. gondii* burden in the brain but did result in increased activation of nuclear factor kappa B (NF-κB) pathway and subsequently upregulation of CCL5, accompanied by increased T-cell infiltration and neuronal death^67^. NF-κB activation in astrocytes regulates inflammatory responses involved in pathological outcomes of neurodegenerative diseases (reviewed in 68). For example, activation of astroglia NF-κB leads to overexpression of complement protein C3, reduced synaptogenesis, and disruption of dendrite morphology contributing to impairment of neuronal activity^69^. *In vitro* inhibition of NF-κB in astrocytes exposed to *T. gondii* antigens consistently decreased the levels of neurotoxic markers, including complement protein C3, suggesting that astrocytic NF-κB signaling may contribute to neurological deficits of CT^70^. However, by inducing IL-1, NF-κB signaling itself is critical for instigating TGF-β counter-regulation^71^. Negative feedback regulation by TGF-β may represent an important mechanism for minimizing the detrimental effects of the pro-inflammatory response while maintaining its parasite restrictive functions.

In addition to mediating immunoregulatory TGF-β signaling, astrocytes perform neuroprotective functions, including maintenance of brain water homeostasis and antioxidant defense. Expression of the water channel aquaporin-4 (AQP4) by astrocytes is critical for preventing excessive brain edema during ECM^72,73^. Upregulation of the antioxidant protein neuroglobin by astrocytes during ECM^74^ may also minimize tissue injury.

## Concluding remarks

**Figure 1.**
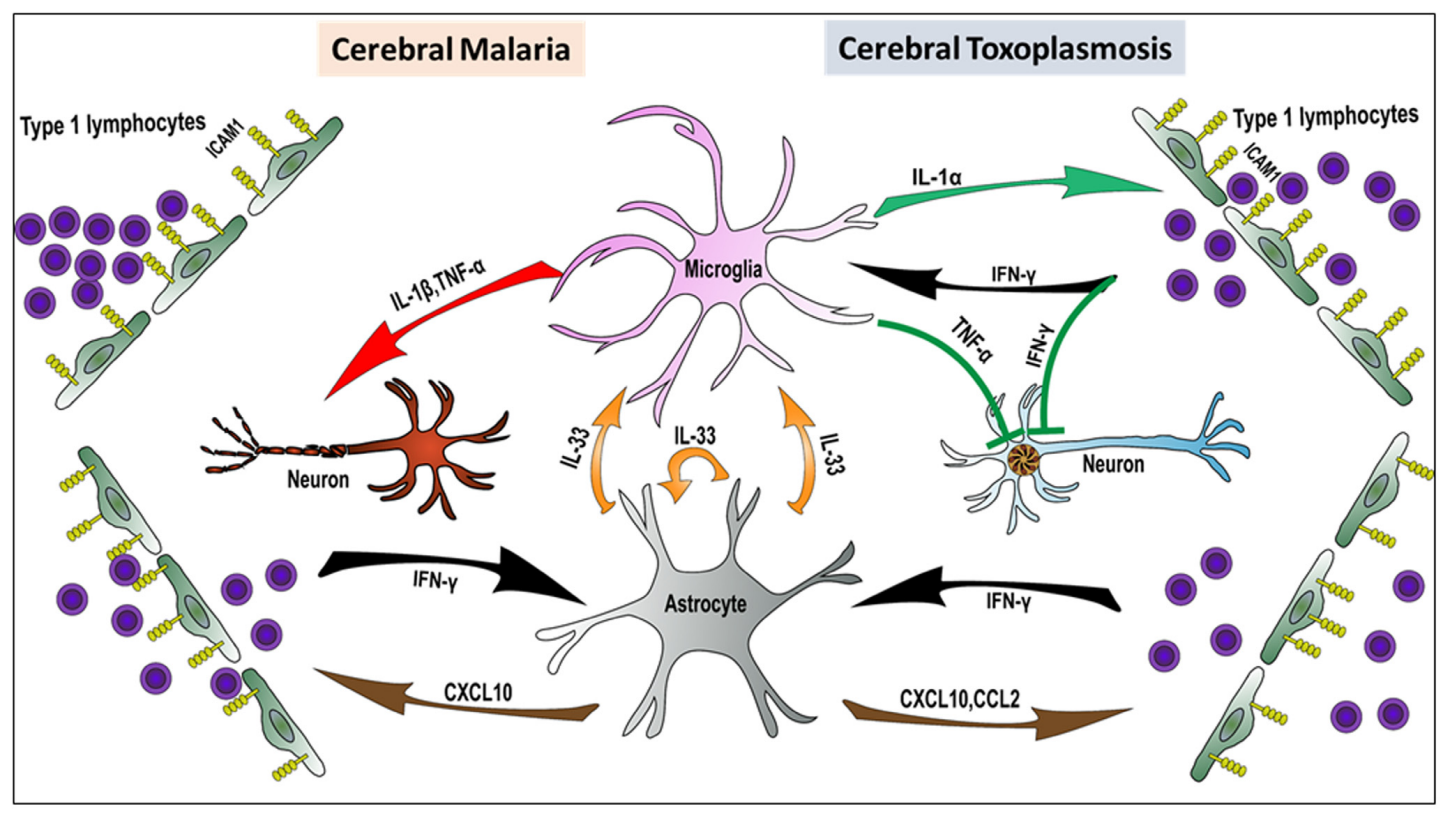
Alarmin-mediated microglia–astrocyte crosstalk during cerebral toxoplasmosis (right) and cerebral malaria (left). Activated astrocytes and microglia initiate signaling pathways required for peripheral immune cell infiltration/sequestration. In cerebral toxoplasmosis, these immune pathways result in the control of *Toxoplasma gondii* replication and partial restoration of tissue homeostasis. Conversely, in cerebral malaria, immune responses elicited in microglia and astrocytes lead to impairment of neuronal function and exacerbation of neurological symptoms. ICAM1, intercellular adhesion molecule 1; IFN-γ, interferon gamma; IL, interleukin; TNF-α, tumor necrosis factor alpha.

Here, we have discussed the experimental evidence indicating that microglia and astrocytes are critical regulators of CNS immune responses during *Toxoplasma* and *Plasmodium* infection. Reflecting the distinct cellular tropisms of these two infections, these immune responses are primarily protective in CT because they drive effector mechanisms critical for controlling *T. gondii* replication in the CNS. By contrast, in CM, the T_H_1 response is largely detrimental, negatively affecting neurological function. However, it is likely that collateral neurotoxic effects occur during CT when the extent of glial activation exceeds what is required for parasite removal. As shown in Figure 1, tissue-resident astrocytes and microglia act as sentinel innate cells that trigger the infiltration/sequestration of the CNS by the peripheral immune cells. Crosstalk between astrocytes (also oligodendrocytes) and microglia is mediated by the alarmins IL-33 and IL-1. It will be important to investigate what parasite-derived versus endogenous signals drive alarmin upregulation and release.

The primary consequence of immune infiltration by type 1 lymphocytes is the differentiation of microglia and astrocytes into M1-like and A1-like type cells^75,76^, that have antiparasitic effector functions but also possess neurotoxic potential. As discussed above, immunoregulation by TGF-β is essential for curtailing this inherently destructive potential. What signals trigger the transitioning of astrocyte and microglial phenotypes from neurotoxic to reparative is another area for future studies. Deployment of single-cell RNA-sequencing technology will be helpful in defining the cellular heterogeneity, dynamics, and regulatory circuits that govern this important transition.
